# Inability to accumulate Ni in a genus of hyperaccumulators: the paradox of *Odontarrhena sibirica* (Brassicaceae)

**DOI:** 10.1007/s00425-020-03507-x

**Published:** 2020-11-10

**Authors:** Isabella Bettarini, Ilaria Colzi, Cristina Gonnelli, Luigia Pazzagli, Roger D. Reeves, Federico Selvi

**Affiliations:** 1 Department of Biomedical Experimental and Clinical Sciences, University of Firenze, Viale Morgagni 50, 50134 Firenze, Italy; 2 Department of Biology, University of Firenze, Via G. La Pira 4, 50121 Firenze, Italy; 3 Palmerston North, 4410 New Zealand; 4 Department of Agriculture, Food, Environment and Forestry, Laboratories of Botany, University of Firenze, P. le Cascine 28, 50144 Firenze, Italy

**Keywords:** *Alyssum*, Cruciferae, Herbarium specimens, Metal accumulation, Serpentine plants, Ultramafics

## Abstract

**Main conclusion:**

*Odontarrhena* is a highly diverse genus of Ni-hyperaccumulators. Here, we demonstrate substantial inability to accumulate Ni in the facultative serpentinophyte *O. sibirica*, which seems a unique case among the numerous species of the genus that grow on ultramafic soils.

**Abstract:**

*Odontarrhena* is the most diverse genus of Ni-accumulating plants in W Eurasia, with most taxa growing obligatorily or facultatively on ultramafic soils. A notable exception may be *O. sibirica*, a facultative serpentinophyte from the E Mediterranean and W Asia in which accumulation ability is still enigmatic. We addressed this issue using observational and experimental methods. Atomic Absorption Analysis of 33 herbarium specimens and plant and soil samples from seven ultramafic and non-ultramafic sites in Greece revealed shoot Ni values always much lower than 1000 µg g^−1^, non-significant differences between plants from the two soil types and no relationship with soil pH. Only two Turkish specimens from waste mines had shoot Ni concentration > 1000 µg g^−1^. The reasons for this deviating result remain obscure, but may be associated with inherent peculiarities of the local populations. When cultivated together with congeneric Ni-accumulating species on the same natural ultramafic soil, only *O. sibirica* was unable to accumulate the metal. Although plant growth was stimulated in hydroponics at relatively low NiSO_4_ levels (50–150 µM), as typical for hyperaccumulators, Ni-accumulation occurred only at higher concentrations which had a toxic effect. This peculiar combination of Ni-response traits could be the result of a partial evolutionary loss of ability with respect to all other Ni-accumulating congeneric species. For this, *O. sibirica* could represent a unique model system for further studies on the evolutionary dynamics, physiological mechanisms and genetic control of metal accumulation and homeostasis.

**Electronic supplementary material:**

The online version of this article (10.1007/s00425-020-03507-x) contains supplementary material, which is available to authorized users.

## Introduction

Nickel (Ni) is a plant micronutrient required at very low concentrations of about 0.5–10 µg g^−1^ DW and present in the enzymes urease and glyoxalase I (Brown et al. 1987; Chen et al. 2009; Boer et al. 2014). Although concentrations above 10–50 µg g^−1^ DW are considered toxic (Yusuf et al. 2011), a small group of plants, known as Ni hyperaccumulators, are able to store more than 1000 µg Ni g^−1^ DW in their leaf tissues without any toxicity symptoms (Reeves 1992; van der Ent et al. 2013). Several physiological mechanisms contribute to Ni hyperaccumulation, whose function is most probably plant protection against herbivores or pathogens because of the toxic effect of high metal concentration in shoots (Martens and Boyd 2002; Palomino et al. 2007; Boyd 2012). Such mechanisms are thought to cause the higher basal requirement of the metal displayed by some Ni-plants, its enhanced sequestration and compartmentation in leaves, associated with a lower efficiency in its metabolic use (Hörger et al. 2013; Cappa and Pilon-Smits 2014; Deng et al. 2017 and references therein).

According to Reeves et al. (2018a, b), Ni accumulation ability is possessed by 532 species of 53 families and 130 genera of Eudicot angiosperms that occur on nearly all Earth’s continents (see also van der Pas and Ingle 2019). Given the lack of phylogenetic affinity between most of these groups, it evolved independently in different ancestral lineages and distant regions of the world (Macnair 2002; Krämer 2010). The great majority of Ni-hyperaccumulating species are found on metalliferous soils derived from ultramafic rocks that are naturally rich in this and other trace metals like chromium and cobalt (Brooks 1987). Although most of these species are restricted to ultramafic soils, with no chance of growing on other types of substrate, some others are facultative and can grow on non-metalliferous soils as well (Pollard et al. 2014). Facultative species are especially useful for investigating adaptation to ultramafic soils and micro-evolution of Ni-tolerance and accumulation ability, as they offer the opportunity to compare conspecific populations exposed to different levels of this trace metal (Pollard et al. 2014).

Accumulation of nickel was first discovered in the Italian endemic *Odontarrhena bertolonii* (Desv.) Jord. and Fourr. (syn. *Alyssum bertolonii* Desv.; Minguzzi and Vergnano 1948), a species of the most diverse and widespread group of hyperaccumulators in Europe and west Asia, the genus *Odontarrhena* C.A.Mey. ex Ledeb.[= *Alyssum* L. subgen. *Odontarrhena* (C.A.Mey. ex Ledeb.) W.D.G.Koch; Brooks et al. 1979; Reeves et al. 1983]. *Alyssum* and *Odontarrhena* are two separate genera of the tribe Alysseae without direct affinity to each other (Warwick et al. 2008; Cecchi et al. 2010, 2013; Rešetnik et al. 2013). Remarkably, no Ni-hyperaccumulator species are known in *Alyssum*, despite the fact that a number of them can grow on ultramafic soils (e.g., *A. vourinonense* Dudley and Rechinger and *A. montanum* L.; Reeves et al. 1983; Selvi 2007). Based on their soil preferences, the ca. 90 species of *Odontarrhena* (Španiel et al. 2015; AlyBase, https://www.alysseae.sav.sk/) can be grouped into three major categories: (i) obligate serpentinophytes, often endemic to outcrops of more or less restricted geographic areas, (ii) facultative serpentinophytes, including populations from ultramafic and non-ultramafic soils, and (iii) non-serpentinophytes, occurring only on non-ultramafic soils, though often rich in Mg, as for example dolomite. Based on present-day knowledge, all taxa in the genus belonging to the first category are “obligate hyperaccumulators” (sensu Brooks et al. 1979; Pollard et al. 2002), while the second category is that of “facultative hyperaccumulators” (sensu Pollard et al. 2014; Reeves et al. 2015). In these species, only the populations from ultramafic soils hyperaccumulate Ni in their leaves, while those from other soil types with low Ni concentration do not [i.e. the W Mediterranean *O. serpyllifolia* (Desf.) Jord. and Fourr. investigated by Morrison et al. (1980); or some Iranian taxa investigated by Ghaderian et al. (2007a, b)]. Regarding the third category, it is still unclear whether the species that never grow on ultramafic soil can accumulate Ni when their growth substrate is artificially enriched with this metal, as no experimental studies have been conducted to date.

Among the obligate and facultative serpentinophytic species of *Odontarrhena* that have been investigated so far, however, one exists in which shoot Ni concentrations in plants from ultramafic soils was mostly found to be lower than the threshold of 1000 µg g^−1^. This is *O. sibirica* (Willd.) Španiel, Al-Shehbaz, D.A.German and Marhold (syn. *Alyssum sibiricum* Willd.), a facultative serpentinophyte widely distributed from the Balkans to Russia and W Asia (Reeves and Adıgüzel 2004; Reeves et al. 2015; Global Hyperaccumulator database; https://hyperaccumulators.smi.uq.edu.au/collection/). Phylogenetically, this species is included in the O3 clade of *Odontarrhena*, where it is sister to a group of mainly non-serpentine and non-hyperaccumulator species from mountain habitats of the Alps, Balkans and Sicily [i.e. *O. alpestris* Ledeb., *O. borzaeana* (Nyár.) D.A. German and *O. nebrodensis* (Tineo) L.Cecchi and Selvi; Cecchi et al. (2010)]; other species in the same clade, however, are serpentine endemics with remarkable accumulation ability, such as the Cyprus serpentine endemic *O. troodi* (Boiss.) Španiel, Al-Shehbaz, D.A.German and Marhold (Reeves et al. 1983). In *O. sibirica*, Brooks et al. (1979) and Morrison (1980) reported shoot Ni concentrations of < 25 µg g^−1^ in accessions from unknown soil types, and a value of 487 µg g^−1^ in a specimen from an ultramafic location in northern Greece. Reeves and Adıgüzel (2008) analysed 21 samples from Turkey and found a median of 132 µg g^−1^ and a range of variation as wide as < 1–8810 µg g^−1^. Remarkably, Ni concentration in 16 accessions from ultramafic soil was found to fall short of the 1000 µg g^−1^ mark, and only five had Ni in leaves at 2160–8810 µg g^−1^. Because of this, *O. sibirica* is currently regarded as a facultative Ni hyperaccumulator but further studies are deemed necessary to discover the causes of such contrasting data (Reeves et al. 2015; Global Hyperaccumulator Database). Actually, this species offers a unique opportunity to examine whether Ni accumulation in serpentinophytic taxa of genus *Odontarrhena* can be “erratic” or even largely lacking, which would be a new finding in both cases. Erratic accumulation is a rare phenomenon that was observed in the Australian obligate endemic *Pimelea leptospermoides* (Thymelaeaceae; Reeves et al. 2015) and may depend on inherent differences between populations and/or local soil factors affecting Ni-availability in the soil, especially pH. In the case of *P. leptospermoides*, plants from more acid soils had considerably higher Ni concentrations, and this factor was believed more likely to determine erratic accumulation than “inherent” differences among populations. Although this mechanism was supposed to explain differences between accessions of also *O. sibirica* (Reeves et al. 2015), the hypothesis that Ni accumulation may be due to inherent differences between populations is still to be investigated. If supported, this would pose the relevant question of the nature of these differences or the causes of such inability, making of *O. sibirica* a unique model system to study the physiological mechanisms, genetic bases and evolutionary dynamics of metal accumulation ability in *Odontarrhena* and Brassicaceae, given its phylogenetic affinity to a multitude of congeneric Ni-accumulating taxa.

Using an observational and experimental approach based on herbarium specimens, natural populations and plants in controlled conditions, we could bring more light into the question of Ni-accumulation ability in this species and provide new data for the development of the two above hypotheses (differences between plant populations or different soil conditions).

## Materials and methods

### The target species

*Odontarrhena sibirica* is a low (up to 20 cm) perennial herb with numerous non-flowering procumbent stems from a woody base; these bear orbicular-spathulate leaves densely covered on both surfaces with whitish pubescence of stellate hairs; the fertile stems produce a broadly corymbose inflorescence of bright yellow flowers borne in numerous partial racemes; the fruits are obovate siliculae S-shaped in cross section, with asymmetrically inflated valves covered with stellate hairs outside. The species range spans from the SW Balkans, especially Greece, to east Turkey, Crimea and possibly further east in Russia (Ball and Dudley 1993; Hartvig 2002). However, the taxonomy and the range of the species are still not fully understood. It grows on maritime sands, gravelly river beds, open sandy or gravelly places in phrygana, and scrub and oak forest, from sea level to 800 m a.s.l. (Hartvig 2002). Though the species can grow on a variety of soil types, in Turkey and the Balkans, it is often found on limestone or ultramafic rocks, especially serpentinite. Intraspecific phenotypic variation is broad but not clearly connected to soil type (Hartvig 2002 and pers. obs. in plants from Greece and Turkey).

### Plant material

#### Herbarium specimens

For the observational part of this study, we first analysed material from 33 specimens kept in the Herbaria B (3), E (3), FI (14), G (9) and W (4; acronyms according to *Index Herbariorum* https://sweetgum.nybg.org/science/ih/). We based our selection on the quality of the specimens (collection age, abundance of material, conservation status), on their geographic origin and on the type of soil as reported on the label of the herbarium sheet. In doing this, we tried to obtain a balanced geographic and ecological sample, including material from Greece and Turkey and from typical ultramafic (*U* = 9 specimens) and non-ultramafic soils (NU = 11); only four specimens from Turkey were unknown for the soil type (referred to as “uncertain”). Six and three specimens from, respectively, the areas of Corinth and Grevena (Greece) were not from a typical ultramafic soil, but this was still high in Ni (> 1000 µg g^−1^) because enrichment by serpentine material from adjacent ultramafic outcrops (Supplementary Table S1 and Results); for statistical comparisons, these were placed in a separate group (NU-HNi = 9). To check the soil type in the gathering localities of the 9 specimens above and in those of two other collections from N Greece (between Konitsa and Eptahori), we visited those sites and collected soil samples as close as possible, based on geographical details or GPS coordinates reported on the label of the herbarium sheet. Importantly, we could also re-analyse two of the five Turkish specimens (herbarium collections by R.D. Reeves no. 2043 and 2056) with Ni > 1000 µg g^−1^ in Reeves and Adıgüzel (2008). This allowed to exclude species identification errors, which have occasionally caused confusion in previous metal accumulation reports.

The full list of analysed specimens is given in Supplementary Table S1. We collected an amount < 0.08 g of dry shoot tissue from different parts of each herbarium specimen for analyses as described below.

#### Native populations

The study of herbarium material was integrated with the analysis of plants and soil samples from native populations collected in Greece in August 2018 and June 2019. These were from three typical ultramafic sites (*U*), two non-ultramafic sites (NU) and two mixed ultramafic-non-ultramafic sites with high Ni (NU-HNi); codes, geographical details and soil type of the sampled populations are given in Table 1.

**Table 1:** Geographical details and voucher specimens of the native populations of *Odontarrhena* in Greece sampled for the purposes of this study

Taxon and Site	Code	Lat Long	Alt (s.l.m.)	Soil type	Voucher
*O. sibirica* (Willd.) Španiel et al					
W Makedonia, Konitsa to Eptahori before junction to Arrenes	*Os1*	40°13′ N 20°58′ E	790	Ultramafic	*Bettarini* and *Selvi s.no.* 03.06.2019, FI
Corinth, Loutraki, Corinth isthmus	*Os2*	37°57′ N 22°57′ E	1	Sandy-gravelly with serpentine	*Bettarini* and *Selvi* FI056346
Evrou, Soufliou, around Dadia	*Os3*	41°10′ N 26°12′ E	240	Ultramafic	*Bettarini* and *Selvi* FI055811
Grevenon, Grevena, ca. 1 km S of Eleftherohori	*Os4*	40°01′ N 21°29′ E	760	Sandy-gravelly conglomerate with serpentine and schist	*Bettarini* and *Selvi* FI056363
Euboea, ca. 1 km S of Limni	*Os5*	38°45′N 23°19′ E	2	Ultramafic	*Bettarini* and *Selvi* FI056353
Konitsa to Eptahori, near junction to Langada	*Os6*	40°13′N 20°50′E	680	Schist	*Bettarini* and *Selvi* FI056376
Serres, Sidirokastro towards Kapnofito	*Os7*	41°19′ N 23°29′ E	590	Llimestone	*Bettarini* and *Selvi* FI056380
*O. chalcidica* (Janka) Španiel et al					
Epirus, Metsovo near Malakasi	*Oc1*	39°46′ N 21°18′ E	860	Ultramafic	*Bettarini* and *Selvi* FI055798
Chalkidiki peninsula, between Hierisso and Gomati	*Oc2*	40°23′ N 23°48′ E	150	Schist	*Bettarini* and *Selvi* FI055805
*O. muralis* (Waldst. & Kit.) Endl					
Makedonia, 3 km N of Paranesti	*Om1*	41°20′ N 24°26′ E	260	Granite	*Bettarini* and *Selvi* FI055807
*O. smolikana* (Nyár.) Španiel et al					
Epirus, Mt. Vasilitsas	*Osm1*	40°03′ N 21°04′ E	1800	Ultramafic	*Bettarini* and *Selvi* FI055817

At each location, five adult plants spaced at least 20 m and of comparable size were randomly collected with the whole root system and dried in a herbarium press. In addition, ten bulk soil samples of ca. 20 g were collected at 1–15 cm depth very close to the sampled plants, and then pooled together to obtain a single sample (ca. 200 g), which was then dried at room temperature.

### Experimental tests

#### Hydroponic cultivations

Seeds of the serpentine population of Limni in Euboea (*Os*5, Table 1), were randomly collected from 50 plants on a surface of ca. 3 km^2^. Unfortunately, no fully ripe seeds from the other populations in Greece were available at the time of our field survey (early June).

Seeds were sown in peat soil and 6-week-old seedlings were transferred to hydroponic culture, in 1-L polyethylene pots containing a modified half-strength Hoagland’s solution (Hoagland and Arnon 1950) in milliQ-water (Millipore, Billerica, MA, USA) buffered with 2 mM 2-morpholinoethanesulphonic acid, pH 5.5, adjusted with KOH. Nutrient solutions were changed weekly and plants were grown in a growth chamber (24/16 °C day/night; light intensity 100 µmol m^−2^ s^−1^, 16-h (day) photoperiod; relative humidity 60–65%). After 3 weeks of pre-culture, the length of roots and shoots of each plant was measured before exposing plants to increasing NiSO_4_ concentrations (0, 50, 100, 250, 500, 1000, 2000, 3000 µM, 12 plants per treatment, one plant per pot), in a background solution of the same composition as the pre-culture solution for 7 days. The “0” treatment consisted of Hoagland solution with no addition of NiSO_4_ (Ni concentration < 150 nM) and will hereinafter be referred to as control solution. Next, all plants were collected after measuring the length of roots and shoots to determine their growth increment. For comparison, plantlets of the facultative Ni-hyperaccumulator species *O. chalcidica* (Janka) Španiel, Al-Shehbaz, D.A.German & Marhold (Greek serpentine population *Oc*1, Table 1) were subject to the same experimental procedure.

#### Pot cultivation

To characterize the Ni-accumulation behaviour of *O. sibirica* on natural Ni-rich soil, we performed a common garden experiment including three other *Odontarrhena* species for comparison. These were *O. chalcidica* (two accessions: one serpentine and one non-serpentine), the mainly non-hyperaccumulator *O. muralis* (Waldst. & Kit.) Endl. (see Cecchi et al. 2018; Bettarini et al. 2019), and the obligate Ni-hyperaccumulator *O. smolikana* (Nyár.) Španiel, Al-Shehbaz, D.A.German and Marhold subsp. *smolikana*. Geographical details of the collection locality for each accession are given in Table 1. In this experiment, plants were cultivated in pots filled with natural serpentine soil collected in Tuscany near Roccatederighi (Grosseto province, Italy), with pH = 7.98 ± 0.15, total Ni = 1573 ± 32, Mn = 877 ± 18, Fe = 49,206 ± 1514, Zn = 26.2 ± 0.6, Ca = 5475 ± 343, and Mg = 38,567 ± 3381 (µg g^−1^ DW mean of 5 samples ± SE). Seeds of all species were sown in early December 2019 in a greenhouse, and five plants per species were harvested after 6 weeks to determine Ni, Mn, Zn, Fe, Ca, and Mg concentration in their shoots.

### Soil and plant analyses

Each bulk soil sample from the Greek native populations was sieved with a 2 mm mesh stainless steel sieve and placed in an oven at 50 °C for 7 days (Bettarini et al. 2019). From each sample, five subsamples of about 0.5 g were digested using 10 mL of 69% HNO_3_ in a microwave digestion system (Mars 6, CEM). Nickel concentrations in the digests were determined by flame atomic absorption spectroscopy (AAS) using a PinAAcle 500 (Perkin Elmer, Waltham, MA, USA) and used to calculate metal concentration in the soils. Soil pH was determined by placing 5 g of soil in a beaker with 10 mL of demineralized H_2_O, shaking the solution for 20 s and then waiting for 60 min; pH readings of the resulting slurry were taken with a calibrated Field Scout pH meter (Spectrum Technologies, Inc.), two readings for each sample. The same protocol was applied to characterize the natural soil for the pot cultivation experiment, using two samples from each pot taken at plant harvest.

Herbarium material and plants from the native populations, from the hydroponic cultivation and from the pot experiment were carefully washed for 10 min with milliQ-water and then dried at 50 °C for 48 h (Selvi et al. 2017). Next, aliquots of ca. 0.02 g (herbarium material and hydroponic plants) or ca. 0.05 g (native plants) from each sample were digested and analysed with AAS as described for soils. To determine metals (Ni for all samples; Mn, Fe, Zn, Ca and Mg for the pot cultivated plants), three analytical replicates were taken for each aliquot or sample. Roots from half of the plants from the hydroponics were carefully washed with 10 mM Pb(NO_3_)_2_ at 4 °C for 20 min to desorb metals adhering to the root cell wall, as in Bazihizina et al. (2015). Apoplastic Ni concentration in roots was calculated as the difference between metal concentration in non-desorbed and desorbed samples.

### Data analyses

After determining the frequency of specimens for five main categories of Ni concentration (mean and median values), the four groups based on soil type were compared using the Mann–Whitney *U* test that is suitable for non-parametric datasets (assessed with the Shapiro–Wilk test). Similarly, we compared Ni concentration in soil and native plant samples from Greece, at the population level and grouping populations by soil type. Linear regression was used to fit the relationship between soil pH and Ni concentration and plant Ni concentrations in the samples from the native populations; significance level was set at *P* value < 0.05. For the common garden experiment, the element concentrations determined in shoots were checked for normality by the Shapiro–Wilk test and mean values were compared by one-way ANOVA followed by Tukey post hoc test. Statistical analyses and plot drawing were conducted using Past version 3.23 (Hammer et al. 2001).

For the analysis of the growth response to Ni treatment in hydroponics, due to the stimulating effect of the metal in the low-dose zone, experimental data points were fitted to the Brain–Cousens model (Brain and Cousens 1989). This model, developed to validate the presence of significant hormetic effects of toxicants on organism growth, is necessary for the estimation of the parameters describing the stimulating effect, which is not taken into account by a four-parameter logistic curve. The fitting to the Brain–Cousens model provided the following parameters: the maximum stimulation dose (MSD), the maximum mean response (MAX, used for the calculation of the hormetic magnitude as MAX*100/length in control condition, that we named HM) and the half-maximal effective concentration (EC_50_). The drc package (Ritz et al. 2015) as implemented in R Studio version R 3.4.3 (R Core Team 2017) was used for curve fitting of concentration–response data.

## Results

### Herbarium specimens

**Fig. 1:**
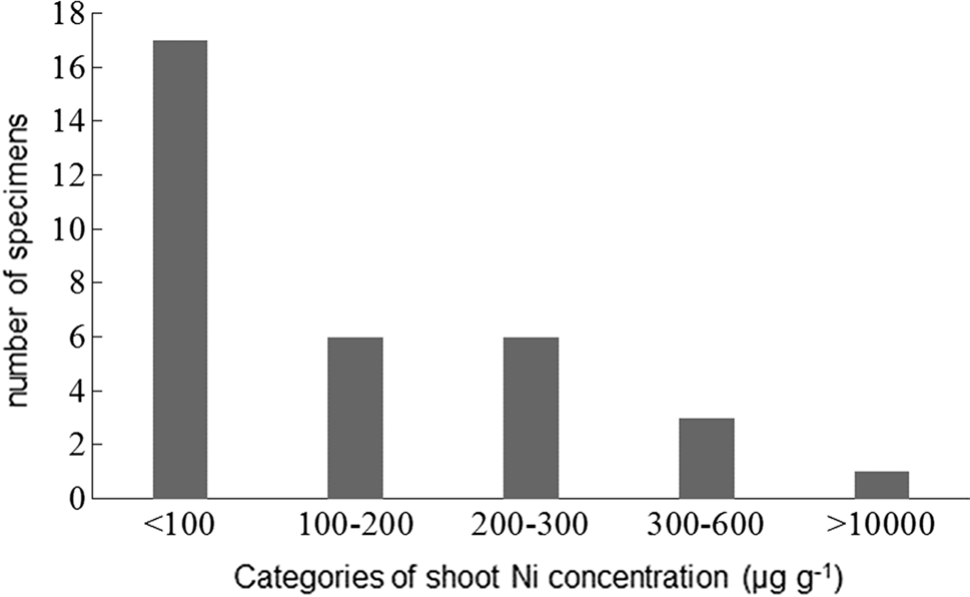
Frequency distribution of 33 herbarium specimens of *Odontarrhena sibirica* across five main categories of shoot Ni concentration (µg g^−1^DW)

**Fig. 2:**
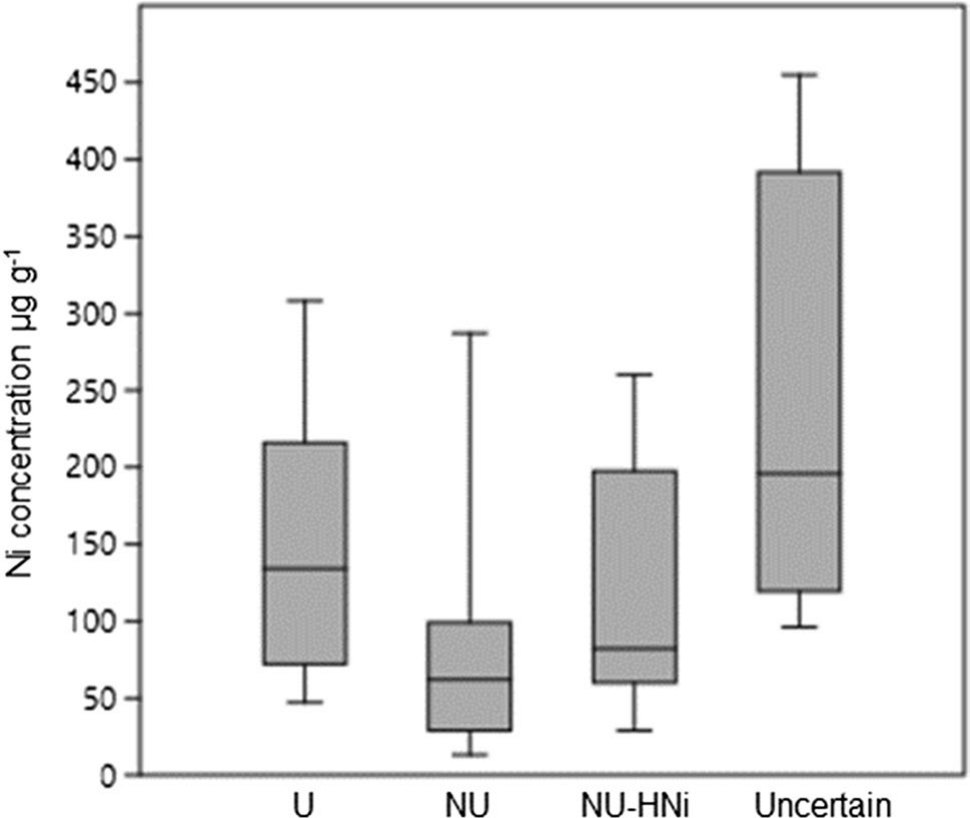
Box-Whisker plot of shoot Ni concentration (µg g^−1^ DW) in herbarium specimens of *O. sibirica* from ultramafic sites (*U* = 6); non- ultramafic sites with high Ni (NU-HNi = 9); non-ultramafic sites with low Ni (NU = 11) and sites with uncertain soil type Ni (uncertain = 4)

Shoot Ni concentration for each of the 33 analysed specimens is given in Supplementary Table S1; values ranged from a minimum of 13 to a maximum of 13,440 µg g^−1^ (mean = 583; median = 99; SD = 2328). The frequency distribution of Ni concentration values was highly uneven, with ca. half of the specimens (17) showing Ni < 100 µg g^−1^; six were included in the range 100–200 and six were between 200 and 300 µg g^−1^ (Fig. 1). Two remarkable exceptions were the accessions from disturbed ultramafic soils around mining sites in the region of Kütahya in W Turkey (herbarium collections by R.D. Reeves no. 2043 and 2056), which showed Ni-concentrations > 1000 µg g^−1^; one of these (Reeves 2056) reached 13,440 µg g^−1^. When excluding these two latter “outliers”, Ni concentration in plants from soils with high Ni (groups U + NU-HNi) was only slightly higher than in those from non-ultramafic soils (NU) and difference was not significant (Fig. 2). The four specimens from uncertain soil type were around 200 µg g^−1^ (both mean and median), suggesting that at least one of them was collected on serpentine soil in Turkey (Nydegger no. 10306 = 455 µg g^−1^).

### Field collected plants

Median values of Ni concentration in the soil of the seven Greek sites ranged from 45 µg g^−1^ in the limestone site in E Macedonia (*Os*7) to over 2100 µg g^−1^ in the mixed serpentine-schist site close to Grevena (*Os*4) and in the *U* site on Euboea (*Os*5); as expected, Ni level was significantly lower in the NU sites (Table 2). Soil pH ranged from 7.35 in a NU site (*Os*6) to 8.22 in the serpentine-enriched site close to Corinth (*Os*2); overall, NU-HNi soils had a significantly higher pH than U soils.

**Table 2:** Soil pH and Ni concentration (µg g^−1^ DW), shoot and root Ni concentration and translocation factor (TF) in *O. sibirica* in natural populations from Greece (codes as in Table 1)

CODE	Soil pH	[Ni] soil	[Ni] shoot	[Ni] root	TF
*Os1*	6.94–7.04 6.99	1413–1610 1595	37–71 59	45–170 58	0.79
*Os3*	7.08–7.07 7.08	1510–1606 1560	163–361 257	90–189 116	1.88
*Os5*	7.61–7.62 7.62	2038–2163 2134	52–110 91	32–148 66	1.36
U	6.94–7.62 7.07 ^a^	1413–2163 1594 ^a^	37–361 94 ^a^	32–189 101 ^a^	1.41 ± 0.20
*Os2*	8.29–8.14 8.22	1493–1816 1549	61–83 70	45–79 58	1.22
*Os4*	7.73–7.92 7.78	2149–2520 2277	265–478 407	52–106 79	5.11
NU-HNi	7.73–8.29 7.98 ^b^	1493–2520 1816 ^a^	61–478 174 ^a^	45–106 72 ^a^	3.16 ± 0.68
*Os6*	7.3–7.4 7.35	197–228 227	22–66 33	16–39 30	1.49
*Os7*	7.63–7.97 7.80	40–47 45	23–56 35	7–37 27	2.65
NU	7.3–7.97 7.51 ^ab^	40–228 122 ^b^	22–66 34 ^b^	7–39 28 ^b^	2.07 ± 0.67

Summary values are given for the three groups of soil type (U = ultramafic accessions; NU = non-ultramafic accessions; NU-HNi = non-ultramafic with high Ni). Values are min and max with medians below; translocation factor is determined as shoot:root ratio (mean of 5 samples ± SE). Letters indicate significant differences between the three edaphic groups for each variable, at *P *value < 0.05

Median values of Ni concentration in shoots varied from 33 in population *Os*6 to 407 µg g^−1^ in population *Os*4; overall the medians for plants from the U and NU-HNi sites were significantly higher than in plants from the NU sites and the same pattern was observed for Ni concentration in roots (Table 2). The translocation factor (shoot:root ratio) ranged from 0.79 (*Os*1) to 5.11 (*Os*4), with no significant differences between the three edaphic groups.

**Fig. 3:**
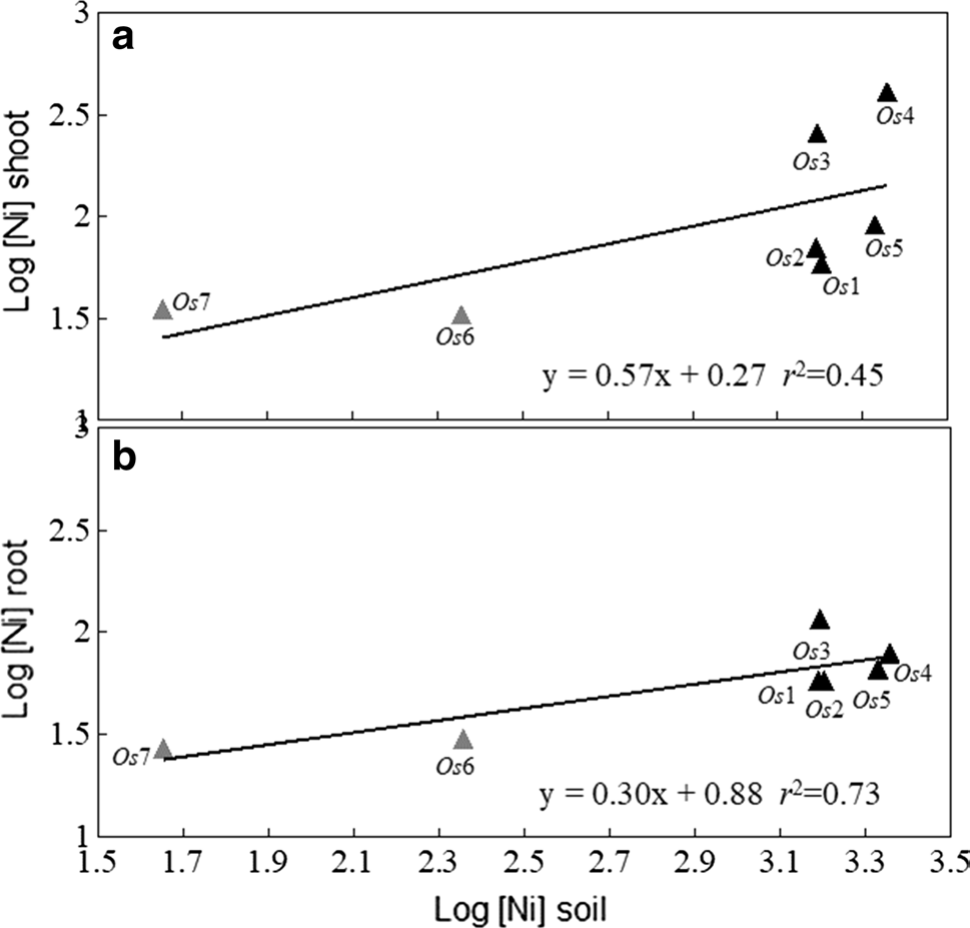
Relationship between Ni concentration in soil and shoots (**a**) and roots (**b**) in *O. sibirica* from Greece; grey triangles are non-ultramafic sites with low Ni content (NU); black triangles are ultramafic and non-ultramafic sites with Ni > 1000 µg g^−1^ (U + NU-HNi); population codes as in Table 1

Ni concentration in both shoots and roots (Fig. 3a, b) tended to increase with soil Ni concentration but this relation was significant for roots only (*P* = 0.014). No significant relationship between soil pH and Ni concentration was detected for roots and shoots.

### Plant growth and Ni accumulation

**Fig. 4:**
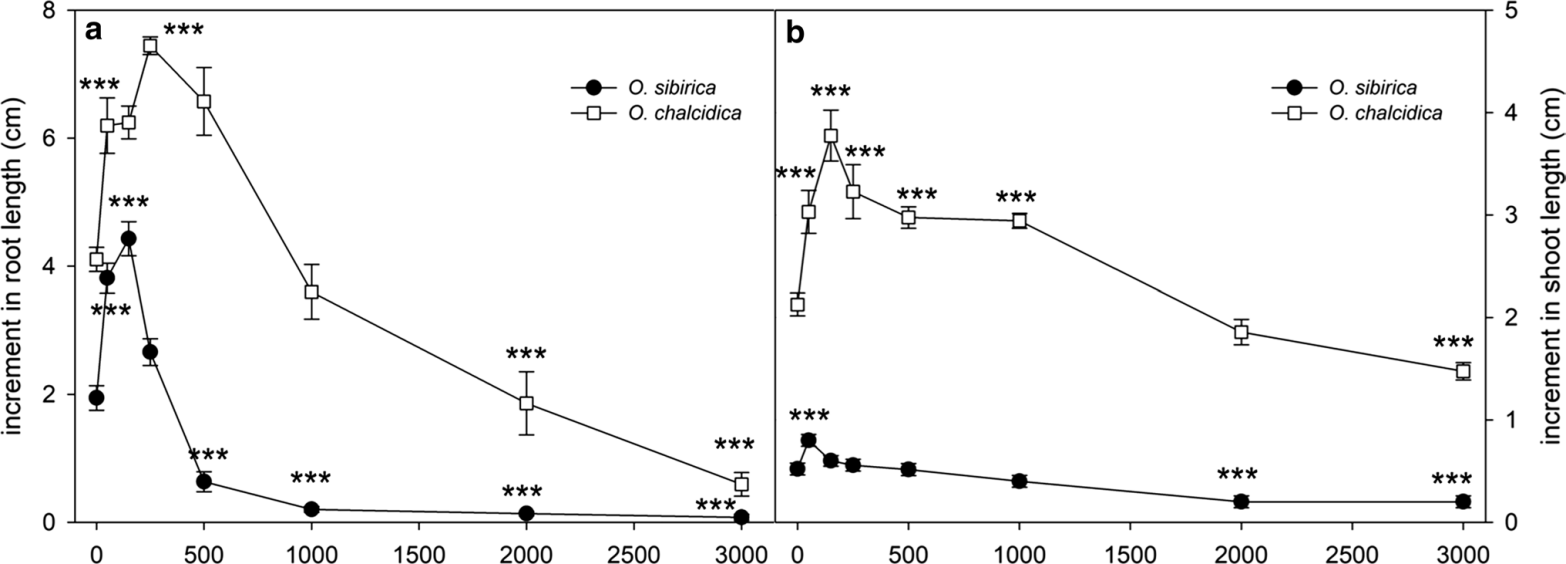
Effect of increasing NiSO_4_ concentrations on root (**a**) and shoot (**b**) growth of *O. sibirica* and *O. chalcidica* after 1 week of treatment. Values are means of 12 samples ± SE. Significant differences, in respect to control solutions, at *P* value < 0.01 are marked with asterisks

Growth of roots and shoots of the serpentine populations of *O. sibirica* and *O. chalcidica* exposed to increasing NiSO_4_ concentrations are shown in Fig. 4 as length increment after seven days of metal treatment. At the lowest concentrations, both plants showed an increased length in respect to the control solution, significant at different treatments depending on species and organ. At higher NiSO_4_ concentrations, there was a clear decline in the growth of both organs, which was highly significant from 500 µM and 2000 µM for root and shoot in *O. sibirica* and from 2000 µM and 3000 µM for root and shoot in *O. chalcidica*.

Due to the initial stimulating effect of Ni on plant growth, data were analyzed by the hormetic model of Brain–Cousens and this provided a significant fitting; the lack-of-fit test gave *P*-values of 0.97 and 0.23 for roots and shoots of *O. sibirica* and 0.24 and 0.26 for roots and shoots of *O. chalcidica*, respectively. The dose–response parameters calculated by the data fitting for roots and shoots, respectively, were: maximum stimulation dose (MSD) = 110.7 and 41.4 µM, maximum mean response (MAX) = 4.82 and 0.79 cm, EC_50_ = 433.3 ± 41.5 and 2151.5 ± 159.3 µM, HM = 260 and 143% for *O. sibirica* and MSD = 292 and 174.5 µM, MAX = 7.39 and 3.57 cm, EC_50_ = 1493.1 ± 205.1 and 3868.9 ± 704.5 µM, HM = 101 and 168% for *O. chalcidica*.

**Fig. 5:**
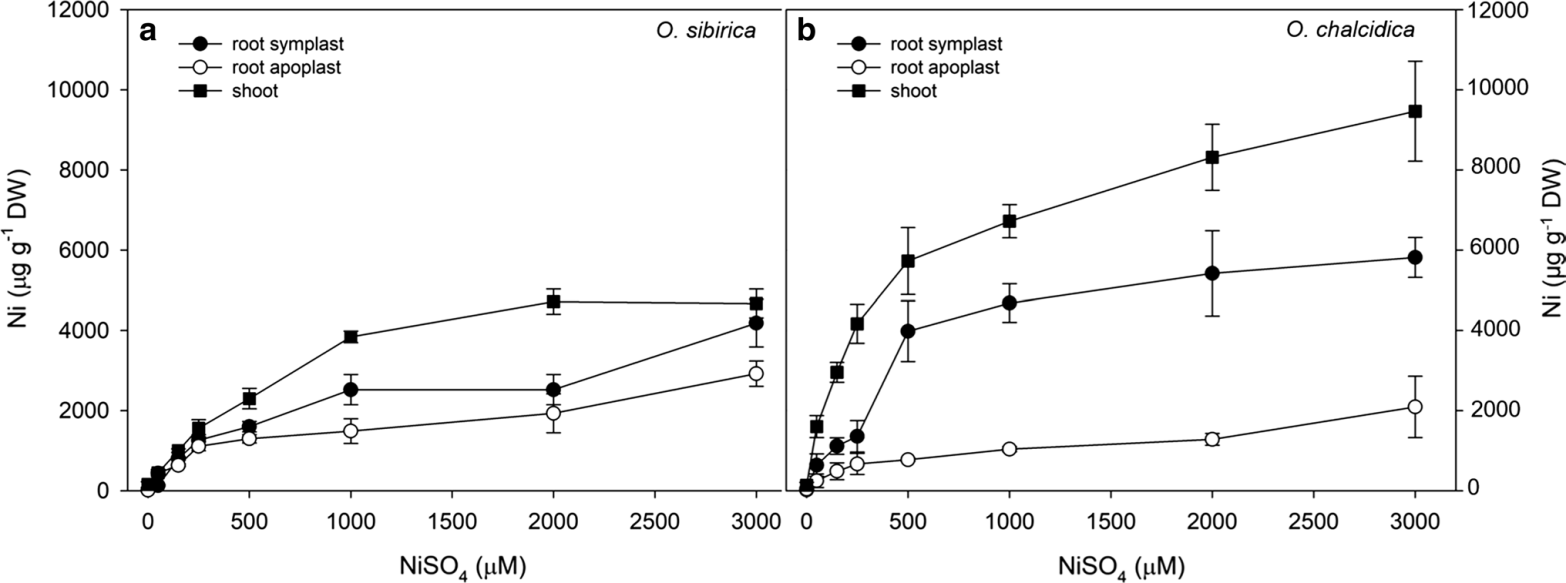
Ni concentration (µg g^−1^DW) in shoots and in apoplastic and symplastic root tissue of *O. sibirica* (**a**) and *O. chalcidica* (**b**) after exposure to increasing NiSO_4_ concentrations for 1 week. Values are means of 12 samples ± SE

Regarding metal accumulation, Ni concentration in roots and shoots of both taxa increased with increasing Ni concentration in the growth substrate (Fig. 5). The highest concentrations were found in shoots, except in *O. sibirica* at the highest treatment concentration used. Finally, in *O. sibirica*, Ni accumulation in roots was slightly higher in the symplast than in the apoplast at the highest Ni doses, whereas it was always higher in *O. chalcidica*.

### Pot experiment

Plants of *O. sibirica, O. chalcidica*, *O. muralis* and *O. smolikana* were all healthy and showed no visible toxicity symptoms after 6 weeks of cultivation on the same natural serpentine soil. Differences in Ni concentration in these plants were highly significant (*P* < 0.001, *F* = 44.3, Table 3). In shoots of *O. chalcidica*, Ni concentration was over 2000 µg g^−1^ DW, despite the fact that the accession from the Chalkidiki peninsula was from a non-ultramafic site; similarly, concentration in *O. smolikana* was over 1800 µg g^−1^. Values of the bioaccumulation factor were well over 1 in both of these obligate or facultative Ni-accumulating taxa. By contrast, the mean Ni concentration in leaves of *O. sibirica* was only 34 µg g^−1^, and the accumulation factor was ca. 70 times lower. Finally, concentration in *O. muralis* was higher than in *O. sibirica* (*P* < 0.05) but still much lower than the 1000 µg g^−1^ mark.

**Table 3:** Concentration of Ni, Mn, Zn, Fe, Ca and Mg (µg g^−1^ DW) at harvest in shoots and Ni bioaccumulation factor in *O. sibirica* and four other *Odontarrhena* accessions grown for six weeks in pots in the same natural serpentine soil

Taxon	Code	[Ni]	[Mn]	[Zn]	[Fe]	[Ca]	[Mg]	Ni b.f
*O. sibirica*	*Os5*	34 ± 13^b^	21 ± 4^d^	49 ± 10^b^	163 ± 27^a^	30213 ± 1640^a^	15905 ± 1108^a^	0.02
*O. chalcidica*	*Oc1*	2204 ± 206^a^	199 ± 13^a^	68 ± 15^ab^	121 ± 42^a^	36323 ± 3384^a^	19464 ± 3187^a^	1.40
*O. chalcidica*	*Oc2*	2133 ± 113^a^	142 ± 12^b^	103 ± 2^a^	81 ± 19^a^	31412 ± 2882^a^	15399 ± 2820^a^	1.36
*O. muralis*	*Om1*	456 ± 30^b^	80 ± 8^c^	81 ± 12^ab^	61 ± 1^a^	39669 ± 3877^a^	18618 ± 1904^a^	0.29
*O. smolikana*	*Os1*	1888 ± 247^a^	171 ± 2^ab^	63 ± 7^ab^	118 ± 16^a^	38242 ± 1431^a^	18636 ± 1099^a^	1.20

Values are means of 5 samples ± SE. Letters indicate significant different groups at *P* value < 0.05

Concerning the other elements in the shoots at the end of the cultivation experiment, differences among taxa were negligible for Fe, Ca and Mg concentrations (*P* > 0.05). Significant divergence was instead found for Mn and Zn. Manganese concentrations were significantly lower in *O. sibirica* and *O. muralis* as compared to the other taxa (*P* < 0.01), with *O. sibirica* showing the lowest mean value. This species showed also the lowest mean value of Zn concentration (*P* < 0.05).

## Discussion

Over 87% of our samples of *O. sibirica* from ultramafic (*U*) and non-ultramafic (NU) sites in Greece and Turkey, including both herbarium specimens and native populations, showed shoot Ni concentrations < 300 µg g^−1^, hence much lower than the 1000 µg g^−1^ threshold that represents the minimum limit for Ni-hyperaccumulation (Reeves 1992; Reeves et al. 2017). Moreover, no significant differences could be found between plants from ultramafic and non-ultramafic sites in samples from natural populations in Greece. Our results are in line with Brooks et al. (1979) and Morrison (1980), who reported, respectively, shoot Ni concentrations < 25 µg µg g^−1^ in accessions from unknown soil types, and a value of 487 µg g^−1^ in a specimen from an ultramafic location in northern Greece. Similarly, our data are close to the median value of 132 µg g^−1^ found by Reeves and Adıgüzel (2008) in 21 accessions from central and western Turkey. Based on these findings, *O. sibirica* seems largely incapable of shoot Ni-accumulation in natural conditions, despite its evident ability to thrive on ultramafic soils. The positive relationship between Ni concentration in the soil and in the plant was significant only for roots, unlike in the hyperaccumulating taxa of *Odontarrhena* from the Balkans (Bettarini et al. 2019). These taxa showed much higher translocation factors (shoot:root ratios of Ni concentration) than *O. sibirica* from sites with high Ni (mean 7.5 ± 0.55 vs. 2.06 ± 0.32; *P* < 0.001), suggesting in the latter species, a different pattern of metal translocation associated with the non-significant relation between soil and shoot Ni concentration. More evidence came from the common garden experiment on natural serpentine soil. While the two hyperaccumulators *O. smolikana* and *O. chalcidica* concentrated the metal in their shoots up to ca. 2000 µg g^−1^ (both the serpentine and, interestingly, the non-serpentine accession in the case of the latter species), *O. sibirica* was totally unable to accumulate it, although the accession used for this experiment was from a typical ultramafic site. *Odontarrhena muralis* was also unable to accumulate the metal, in line with its clear preference for non-serpentine soils. This confirms that most literature reports on Ni-accumulation ability in “*O. muralis*” are to be referred to closely related Ni-accumulating taxa that are widespread in the Balkans, such as *O. chalcidica* and *O. decipiens* (Nyár.) L.Cecchi and Selvi (Cecchi et al. 2018; Bettarini et al. 2019).

The shoot concentration of the other elements analysed was consistent with the typical values known for serpentine plants, with Ca higher than in the substrate and lower Fe, Mg and Mn (Brooks 1987; Kazakou et al. 2008). Compared with the other taxa, however, Mn concentrations were distinctly lower in *O. sibirica* and, to a lesser extent, in *O. muralis*. Actually, the presence of Mn was found to affect Ni accumulation in some hyperaccumulating species of *Odontarrhena* (Broadhurst et al. 2009), suggesting that the two ions could follow, at least in part, the same pathway for uptake, translocation and/or storage. Therefore, plants unable to accumulate Ni would also have lower amounts of Mn, as in the present case. A similar scenario could be assumed for Zn, due to its competition towards Ni in hyperaccumulators (Deng et al. 2014), though *O. muralis* did not show significantly lower levels as *O. sibirica* did, possibly due to its slightly higher ability to accumulate Ni.

In their study, however, Reeves and Adıgüzel (2008) found that five accessions of *O. sibirica* from Turkish ultramafic sites had Ni in leaves at 2160–8810 µg g^−1^. Re-examination of two of these accessions confirmed high shoot Ni concentration, which reached over 13,000 µg g^−1^ in one of them. Unfortunately, the lack of data on the soils where these specimens were collected makes it difficult to elucidate the causes of such unusually high Ni concentrations. According to Reeves et al. (2015), soil pH can strongly influence Ni availability and can explain variability in some facultative hyperaccumulators, such as the Australian *Pimelea leptospermoides*. In this species, higher shoot Ni concentrations were found in plants from sites with soil pH < 6.5, possibly due to an increased availability of Ni consequent to soil acidification. Increased phytoavailability of Ni under artificial cover of *Pinus pinaster* Ait. on serpentine soil was also found by Selvi et al. (2017), who observed a higher shoot/root ratio in plants of *O. bertolonii* associated with a lowering of pH induced by the conifer litter. According to Reeves et al. (2015), variations in soil pH and/or Ni-availability may explain also the erratic Ni accumulation observed in *Evolvulus alsinoides* (L.) L. (Convolvulaceae) and *Hybanthus enneaspermus* (L.) F.Muell. (Violaceae), two widespread species that have been reported with high Ni on serpentine at Ussangoda in Sri Lanka (Rajakaruna and Bohm 2002), but with low Ni in serpentine sites in Queensland and New South Wales, Australia. Remarkably, increasing soil pH was experimentally found to induce a slight decrease of shoot Ni concentration in two typical Ni-accumulating species of *Odontarrhena* cultivated on serpentine soil from Oregon chemically modified to produce a pH range from 5.8 to 7.8 (Kukier et al. 2004). In field-collected plants, however, we found no relationship between soil acidity and Ni concentration in shoots and roots, although the range of pH in our soil samples with high Ni (U and NU-HNi) was relatively large (6.94–8.29). In contrast with recent findings in accumulating *Odontarrhena* taxa from Albania (Bettarini et al. 2019), this result further supports a different behavior of *O. sibirica* in respect to the other serpentinophytic taxa of this genus. Additional evidence came from the hydroponic cultivation experiment with pH solution of 5.5 and full availability of increasing Ni concentrations. Plants did not show any visual symptoms of toxicity in such artificial condition of acidity, and the dose–response curves revealed a significant Ni-induced stimulating effect on growth of both roots and shoots exposed to NiSO_4_ concentrations of 50 and 150 µM (roots only). Since concentrations of this micronutrient even below 5 µM are reported to severely inhibit growth of crop plants (Marschner 1995), detection of positive effect at values ten to thirty times higher than this threshold was unexpected. This indicates tolerance to higher concentrations in respect to normal plants, though accumulating species of *Odontarrhena* have a higher basal requirement for Ni (Krämer et al. 1996; Küpper et al. 2001; Galardi et al. 2007). In *O. sibirica*, the model descriptors (MDS and HM) indicated not only that root growth benefits from higher Ni concentrations in the culture medium than shoots but also that the hormetic effect in roots was stronger than in shoots, for reasons still to be clarified. The differences in the EC_50_ values also revealed that shoots are more tolerant than roots, which, coupled with their higher metal concentrations (see below), is a typical trait of the hyperaccumulator phenotype, usually associated with efficient mechanisms of detoxification of the shoots themselves (Deng et al. 2017). At the highest Ni concentrations not inducing toxic effects on growth, *O. sibirica* was able to express two typical traits of accumulating plants, i.e. the Ni concentrations higher in shoots than in roots and above the 1000 µg g^−1^ threshold in shoots. On the other hand, in the typical hyperaccumulator *O. chalcidica*, shoot Ni concentrations and shoot: root ratio in plants cultivated in the same experimental conditions were distinctly higher than in *O. sibirica* (for example, for plants treated with 250 µM NiSO_4,_ that was the highest non-effective dose in *O. sibirica*, shoot Ni ca. 4100 vs. ca. 1500 µg g^−1^ in *O. sibirica*; mean shoot:root ratio = 3.03 vs. 1.2 in *O. sibirica*), whereas root symplast showed a similar concentration (ca. 1200–1300 µg Ni g^−1^ at 250 µM NiSO_4_). The Ni concentration in the root apoplast was instead higher in *O. sibirica* than in *O. chalcidica* (i.e. 1100 vs. 670 µg g^−1^ at 250 µM NiSO_4_), suggesting a contrasting pattern of root metal allocation that could affect the different uptake and translocation capacity in the two species. In *O. chalcidica*, moreover, the MSD was detected at Ni concentrations nearly three times higher in roots (292 vs. 110.7 µM) and four times higher in shoots (174.5 vs. 41 µM) than in *O. sibirica* and the EC_50_ was higher (1493 vs. 433 µM in roots and 3869 vs. 2151 µM in shoots), thus suggesting a superior Ni tolerance in the real hyperaccumulator *O. chalcidica*. Hence, the hydroponics experiment showed that *O. sibirica* can be forced to express some Ni-accumulation ability in conditions of full metal availability, but at a much lower level as compared to the standard of real hyperaccumulation. In any case, this potential feature appears unlikely to be expressed in natural conditions because the main factor that can increase Ni availability in serpentine soil, that is pH lowering, appeared to cause indirect injury to the plants on ultramafic soils.

Under these circumstances, the hypothesis of inherent differences between the population samples analyzed here and the accumulating specimens from Turkey (Reeves and Adıgüzel 2008) gains support. Such differences may also involve genetic traits, as in the case of *Senecio coronatus* Harv. from S Africa (Asteraceae). In this species, large variation in the Ni-accumulating phenotype and root ultra-structure between hyperaccumulating and non-accumulating populations persisted in plants grown on a common soil and had a genetic basis (Mesjasz-Przybyłowicz et al. 2007). Accordingly, populations and plants of *O. sibirica* with contrasting levels of Ni in their shoots should be compared for their genetic traits as well, also looking at possible hybridization and introgression with other locally coexisting Ni accumulating species of *Odontarrhena.* Hybridization and genetic admixing are indeed known to occur between some sympatric Ni-accumulating species of this genus in similar habitats of the Balkans, especially in anthropogenic sites with heavy disturbance, and not always associated with detectable phenotypic traits in the plants (Cecchi et al. 2018; Coppi et al. 2020 in press). Though the two Turkish accumulating specimens that we examined did not show clear “hybrid signatures”, these were from heavily disturbed sites and growing near other congeneric Ni accumulators [*O. corsica* (Duby) Španiel, Al-Shehbaz, D.A.German and Marhold, *O. dudleyi* (Adıgüzel and R.D.Reeves) Španiel, Al-Shehbaz, D.A.German and Marhold, and group of *O. muralis*], which might have favoured interspecific gene flow and the origin of introgressive populations.

Summing up, our study provides evidence that the Ni-response of *O. sibirica* is unique in *Odontarrhena*. This species appears as the only in the genus with substantial Ni-accumulation inability when growing in natural ultramafic habitats, despite its partially positive response to the metal at low concentrations in artificial conditions. Such a peculiar combination of response traits could be either the result of a gradual process of active evolution of Ni hyperaccumulation in the various ultramafic occurrences of the species, at different stages in different locations, or alternatively, the outcome of a partial evolutionary loss with respect to all other Ni-accumulating serpentinophytic congeneric species. Based on available phylogenetic evidence (Cecchi et al. 2010), both hypotheses are plausible, since *O. sibirica* belongs to the clade O3 with either non-accumulating taxa from non-ultramafic soils (i.e. *O. alpestris*, *O. borzaeana*, *O. nebrodensis*) or obligate serpentine endemics with accumulation ability, such as *O. troodi*, *O. smolikana* and *O. heldreichii* (Reeves et al. 1983). In both cases, *O. sibirica* appears as a unique model system for further studies on the physiological mechanisms, genetic control and evolutionary dynamics of metal accumulation and homeostasis.

## Author contributions statement

IB and FS designed the work and collected the samples in the field. IB, IC, CG and FS acquired and analysed the data. FS, IB and CG wrote the manuscript. IC, RR and LP critically revised the article. All the authors approved the version of the manuscript to be published.

## Electronic supplementary material

Below is the link to the electronic supplementary material.

Supplementary file1 (DOCX 25 KB)

## Abbreviations

NU: Non-ultramafic sites
NU-HNi: Non-ultramafic sites with high Ni
U: Ultramafic sites
