# Supplementary material for: Inability to accumulate Ni in a genus of hyperaccumulators: the paradox of Odontarrhena sibirica (Brassicaceae)
Source: Planta. 2020 Nov 10;252(6):99. doi: 10.1007/s00425-020-03507-x (PMC7655579; doi:10.1007/s00425-020-03507-x)
Supplement: Supplementary file 1 — Supplementary file1 (DOCX 25 KB) [file 425_2020_3507_MOESM1_ESM.docx]

**Supplementary Table S1**

List of examined herbarium specimens with country of origin, collection locality, vouchers (Herbarium codes follow *Index Herbariorum*), and soil type: U = ultramafic, NU = non ultramafic, NU-HNi = non ultramafic but with high Ni (> 1000 µg g^-1^). Ni-concentration in leaves (µg g^-1^ DW) of each specimen are given as mean ± SE (*n* = number of soil or plant subsamples analyzed); Ni-concentration in the soil of sites approximately corresponding to the collection sites of some herbarium specimens are also given (see footnotes).

| **Country** | **Herbarium specimen/collection locality** | **Collector and voucher** | **soil type** | | **[Ni]**  **shoot** | | **[Ni]**  **soil** | |  |  |  |  |
| --- | --- | --- | --- | --- | --- | --- | --- | --- | --- | --- | --- | --- |
| Greece | Ioanninon, Konitsis, along main road Eptachori-Konitsa, 3 km after turnoff to Molista. Scree on road embankment, 500 m, 40°07'N, 20°47'E | *Strid & Vassiliades* 55292 (G, G00426753) |  | NU | | 72 | | 206 ± 4.1  (*n*=4) | | |  |  |
| Greece | Kastorias, 2 km from Epatachori to Pentalofos. Gravelly road embankment. Flysch. Flowers yellow, 950 m, 40°13'N, 21°03'E | *Strid et al.* 46594 (G, G00426767) |  | NU | | 287 | | 249 ± 1.9  (*n*=4) | | |  |  |
| Greece | Kastorias, 6 km from Eptachori to Nea Kotili. Slopes by roadside, 760 m. Flowers yellow. 40°15'N, 21°01' E | *Tan &* *Vold* 13377 (G, G004267769) |  | U | | 308 | | 1553 ± 47^1^  (*n*=4) | | |  |  |
| Greece | Korinthia, Xylokastro, Krautfluren, Kiesstrand 38°04'09’’N, 22°39'21’’E, 5 m | *Willing* 284.263 (B, B1060037) |  | NU | | 21 ± 6  (*n*=2) | |  | | |  |  |
| Greece | In arenosis maritimis propre Corinthum | *Heldreich* s.no., 29.07.1848 (FI) |  | NU-HNi | | 54.7 ± 4.3  (*n*=3) | | 1547 ± 56.5^2^  (*n*=5) | | |  |  |
| Greece | In regione litorali prope Corinthum ad portum antiquum Loutrakon | *Heldreich* s.no., 21.05.1877 (FI) |  | NU-HNi | | 82 ± 22  (*n*=2) | |  |  |  |  |  |
| Greece | In litore Corinthiaco | *Heldreich* s.no.*,* 4.05.1883 (FI) |  | NU-HNi | | 66.5 ± 4.2  (*n*=4) | |  |  |  |  | |
| Greece | In litore Corinthiaco prope Lutrakum | *Orphanides* 89 (FI) |  | NU-HNi | | 54.7 ± 4.3  (*n*=3) | |  |  |  |  |  |
| Greece | In litore Corinthiaco raro | *Orphanides* s.no. 24.05.1851(FI) |  | NU-HNi | | 29 | |  |  |  |  |  |
| Greece | In Isthmo Corinthiaco inter Loutraki e Calamaki | *Guicciardi,* Herb. Heldreich no. 706 (FI) |  | NU-HNi | | 260 ± 16  (*n*=2) | |  |  |  |  |  |
| Greece | Sterea Ellada, Evvia (Eubea) sul litorale a sud dell'abitato di Limni | *Cecchi & Selvi*, 08.06.2008, no. 08.21 (FI) |  | U | | 72.25 ± 3.4  (*n*=4) | | 2117 ± 27.8^3^  (*n*=4) | | |  |  |
| Greece | Grevenon, by Venetikos river bridge SE of Grevena. Conglomerate rocks & gravel by the river, 520 m, 40°03'N, 21°28'E | *Strid* 49595 (G, G00426755) |  | NU-HNi | | 220 | | 1484 ± 92  (*n*=4) | | |  |  |
| Greece | Nom Grevena, Ep. Grevenon, 1,7 km N Eleftherohori, Hanböschung unter Wiesen, 40°3'N, 21°28'30'' E, 460 m | *Willing* 22.099b (B) |  | NU-HNi | | 119 ± 15  (*n*=2) | |  |  |  |  |  |
| Greece | Nom Grevena, Ep. Grevenon, 1,7 km N Eleftherohori, Hanböschung unter Wiesen, 40°3'N, 21°28'30''E, 460 m | *Willing* 22.099a (B) |  | NU-HNi | | 174.5 ± 3.5  (*n*=2) | |  |  |  |  |  |
| Greece | Nomos Evros, nahe der Strasse von Dadia nach Pessani, ENE der Abzweigung nach Treis Vrysses und Mont Sapka, 330 m Serpentine, Shuttfluren, 41°05'43''N, 26°05'55''E | *Karl* s.no.,10.06.2007 (W) |  | U | | 102 | | 1559 ± 17^4^  (*n*=6) | | |  |  |
| Greece | Evrou, Soufliou. Area called Pessani c.17 km from Dadia to Loutros. Rocky serpentine outcrop surrounded by mixed deciduous woodland, 400 m, 41°06'N, 26°06' E | *Strid et al.* 53079 (G, G00426764) |  | U | | 134 | |  |  |  |  |  |
| Greece | Evrou, Soufliou. 17,5 km from Dadia along road to Loutros. Open, gravelly patches and small stream in deciduous oak woodland, 400m. Serpentine. 41°06'N, 26°06'E | *Strid & Lassen* 50426 (G, G00426756) |  | U | | 135 | |  |  |  |  |  |
| Turkey | In Mt. Sypilo | *Aucher-Eloy, 1848,* no. 293 (FI) |  | NU | | 74.5 ± 5.5  (*n*=2) | |  | | |  |  |
| Turkey | Lydia: Montis Sipyli in regione subalpina et alpina, 9-1200 m | *Bornmüller* 9089 (FI) |  | NU | | 99 | |  | | |  |  |
| Turkey | Lydia: Montis Sipyli, in regione subalpina et alpina | *Bornmüller* 9089 (E, E00373096) |  | NU | | 57 | |  | | |  |  |
| Turkey | Manisa, Spil Dăg, prati rocciosi calcarei poco sotto la cima occidentale, 1300 m | *Brullo et al.* s.no*.*, 23.06.2010 (FI) |  | NU | | 62 ± 4  (*n*=2) | |  | | |  |  |
| Turkey | Denizli, Pamukkale, Bereich der ausgetrackneten Sinterterrassen | *Sorger* 66-6-84 (W) |  | NU | | 232 | |  | | |  |  |
| Turkey | Denizli, Muĝla-Kale, 6 km S Kale, Weizenfeld, 1100 m | *Nydegger* 10306 (G, G00426803) |  | uncertain | | 455 | |  | | |  |  |
| Turkey | Denizli, Tavas, 22 Km nach Tavas, gegen Denizli, Fuss des Honaz Dag, 1150 m | *Huber-Morath* 5577 (G, G00426784) |  | uncertain | | 96 | |  | | |  |  |
| Turkey | Kizilcahaman pendii aridi a ovest del Lago Kurtbogazi, calcare | *Cecchi & Selvi*, s.no., 30.07.2015 (FI) |  | NU | | 30 | |  | | |  |  |
| Turkey | Mudurnu tra Sapanca e Abant oltre Gokcele presso Karacakaya, calcare | *Cecchi & Selvi*, s.no., 24.07.2015 (FI) |  | NU | | 29 | |  | | |  |  |
| Turkey | Eskişehir, ca 10 km a sud-ovest della città lungo la strada n 230 per Kutahya, affioramento collinare di roccia detritica di serpentino, ca 900 m | *Brullo et al.* s.no, 22.06.2010 (Herb. Cecchi 1747, FI) |  | U | | 46.5 ± 5.2  (*n*=4) | |  | | |  |  |
| Turkey | Kütahya: Inegöl – Tavşanlı 14 km S Domaniç, Serpentinfels, 630 m | *Nydegger* 16139 (G, G00426800) |  | U | | 216 | |  | | |  |  |
| Turkey | Kütahya: Tavşanli-Harmançik, 23.5 km W of Tavşanlı, 870 m. Roadside banks and adjacent *Pinus nigra* forest on serpentine, near quarry. 39°38'33"N, 29° 18'E | *Reeves* 2056 (E, E00315433) |  | U | | 13440 ± 953  (*n*=3) | |  | | |  |  |
| Turkey | Kütahya: Tunçbilek near NW edge of opencast coalmine , 1040 m. Ultramafic overburden from mining, ground extensively disturbed, colonized mainly by four *Alyssum* spp. 39°40'33.4"N, 29°25'55.7"E | *Reeves* 2043 (E, E00315430) |  | U | | 1779 | |  | | |  |  |
| Turkey | Asia minor | *Parraiy* s.no. 1844 (FI) |  | uncertain | | 190 | |  | | |  |  |
| Turkey | Asia minor | *Antinori s.no*, Luglio 1869 (FI) |  | uncertain | | 202 | |  | | |  |  |
| Georgia | Tiflis, Festungsfelsen | *Makonsky*, s.no 09.1897 (FI) |  | NU | | 13 | |  | | |  |  |

Value obtained from soil sampled in the site of native populations: ^1^= *Os*1; ^2^ = *Os*2; ^3^ = *Os*5; ^4^ = *Os*3
